# CGGBP1 regulates cell cycle in cancer cells

**DOI:** 10.1186/1471-2199-12-28

**Published:** 2011-07-07

**Authors:** Umashankar Singh, Pernilla Roswall, Lene Uhrbom, Bengt Westermark

**Affiliations:** 1 Department of Immunology, Genetics and Pathology, Rudbeck Laboratory, Uppsala University, 751 85 Uppsala, Sweden; 2 Department of Medical Biochemistry and Biophysics, Karolinska Institute, 171 77 Stockholm, Sweden

## Abstract

**Background:**

CGGBP1 is a CGG-triplet repeat binding protein, which affects transcription from CGG-triplet-rich promoters such as the FMR1 gene and the ribosomal RNA gene clusters. Earlier, we reported some previously unknown functions of CGGBP1 in gene expression during heat shock stress response. Recently we had found CGGBP1 to be a cell cycle regulatory midbody protein required for normal cytokinetic abscission in normal human fibroblasts, which have all the cell cycle regulatory mechanisms intact.

**Results:**

In this study we explored the role of CGGBP1 in the cell cycle in various cancer cell lines. CGGBP1 depletion by RNA interference in tumor-derived cells caused an increase in the cell population at G0/G1 phase and reduced the number of cells in the S phase. CGGBP1 depletion also increased the expression of cell cycle regulatory genes CDKN1A and GAS1, associated with reductions in histone H3 lysine 9 trimethylation in their promoters. By combining RNA interference and genetic mutations, we found that the role of CGGBP1 in cell cycle involves multiple mechanisms, as single deficiencies of CDKN1A, GAS1 as well as TP53, INK4A or ARF failed to rescue the G0/G1 arrest caused by CGGBP1 depletion.

**Conclusions:**

Our results show that CGGBP1 expression is important for cell cycle progression through multiple parallel mechanisms including the regulation of CDKN1A and GAS1 levels.

## Background

CGGBP1 was identified as a CGG triplet repeat binding protein in vitro [1]. Ever since, different studies have focused on its ability to bind to CGG triplet repeats and exert transcriptional repression. Previously, we found that CGGBP1 participates in heat shock stress response by regulating HSF1 expression through heat-sensitive interactions with NFIX and HMGN1 [2,3]. In normal human fibroblasts, which are expected to have all the checkpoints and DNA repair capabilities intact, we recently reported functions of CGGBP1 in cell cycle regulation at the abscission and consequential prevention of tetraploidy [4]. In cancer cells however, which often have various abnormalities in the cell cycle regulatory mechanisms, function of CGGBP1 is unknown and is of obvious interest since loss of cell cycle regulation is an event central to tumorigenesis.

Cell proliferation is tightly regulated by different mechanisms, which can halt it at an appropriate stage of cell cycle in response to abnormalities in extracellular as well as intracellular environment. Physical or chemical stress to the cells, inability to respond to mitogenic signals, trans-mitotic inheritance of polyploidy, DNA damage response or loss of function of critical cell cycle regulatory genes [5-9] exemplify some such conditions that can cause a cell cycle block. The kind of effects these conditions can have on the cell cycle progression could however vary from one cell type to the other depending on their genetic and epigenetic profiles.

Under normal conditions, cell cycle arrest in the G0/G1 phase is associated with the phenomena of quiescence, when cells do not receive enough mitogenic stimulation in terms of growth factors, and senescence, when cells are terminally differentiated and enter a post-mitotic state [10]. Altered expression of critical genes, due to genetic and epigenetic disturbances, can also cause cell cycle disturbances [11-13]. The ability of cells to undergo cell cycle arrest is paramount to the health of any multicellular organism and a complex network of proteins has evolved to execute it. The progression of cell cycle from G1 to S phase is regulated by a well-studied series of events. The cyclin dependent kinases CDK4 and CDK6 must interact with Cyclin D to become active and phosphorylate Rb [14-17]. This phosphorylation of Rb releases it from the transcriptional inhibitory complex with E2F, which then drives the expression of many genes including Cyclin E. Cyclin E complexes with CDK2 to drive entry into S phase. The very first step of this cascade, interactions between CDK4/6 and Cyclin D is inhibited by INK4A and ARF, as they compete with Cyclin D for binding to CDK4/6 [18-20]. Another protein, CDKN1A is a multifaceted regulator of the cell cycle. It inhibits Cyclin E-CDK2 as well as Cyclin D-CDK4 interactions and can arrest cell cycle at G1 or early S phase in response to DNA damage [18-20]. Furthermore, CDKN1A expression is controlled by TP53, a strong tumor suppressor gene activated by DNA damage response, which frequently exhibiting loss of function in many cancers. The mutations in some or many of these cell cycle regulatory genes such as TP53, CDKN1A, INK4A and ARF often underlie the aberrant control of cell cycle and the ability of cancer cells to escape the cell cycle block at G0/G1 phase in response to the stimuli, which would normally cause a G0/G1 arrest. The growth arrest specific gene GAS1 is known to cause G0/G1 arrest in normal as well as transformed cells [21,22] and the mechanisms of the regulation of its levels are less well understood.

In this study, we combined CGGBP1 knockdown by siRNA with different genetic mutations of various cell cycle regulatory genes to rigorously test how CGGBP1 regulates cell cycle in cancer cells. For this we have used well-established human cancer cell lines and also generated new murine glioblastoma cell lines. We report that in all the cell lines examined, CGGBP1 deficiency produced a cell cycle block at G0/G1 phase. Expression analysis of candidate genes showed that CGGBP1 regulates expression of CDKN1A and GAS1 genes. Mutation of CDKN1A and siRNA-mediated depletion of GAS1 could not rescue the CGGBP1 deficiency-induced cell cycle block at G0/G1 phase, suggesting that CGGBP1 controls cell cycle progression through the G1 phase through multiple parallel mechanisms. Our results show new functions for CGGBP1 in cell cycle, in regulation of expression of CDKN1A and GAS1, and provide insights into how CGGBP1 depletion overrides the redundancies of checkpoint escape mechanisms present in different cancer cells.

## Results

### CGGBP1 depletion by siRNA increases G0/G1 phase and reduces S phase cell populations

**Figure 1:**
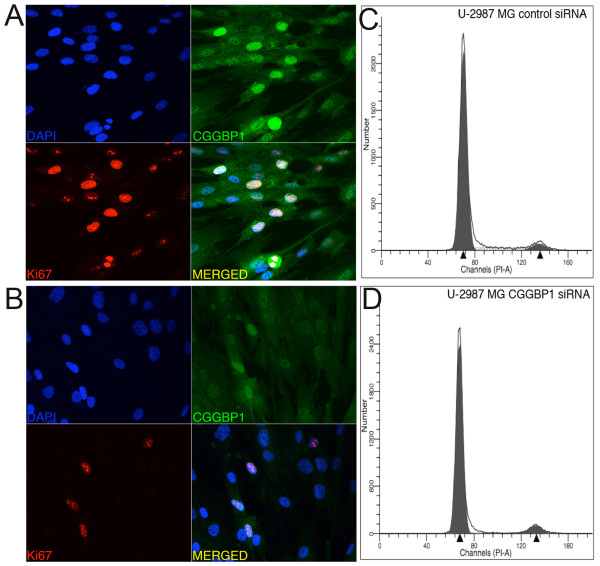
**CGGBP1 siRNA causes decrease in CGGBP1 expression and Ki67 positivity of the nuclei in U-2987 MG cells and blocks cell cycle at G0/G1 phase**. A: Control siRNA, B: CGGBP1 siRNA, C: Flow cytometric pattern of U-2987 MG cells treated with control siRNA, D: Flow cytometric pattern of U-2987 MG cells treated with CGGBP1 siRNA. The strong decrease in the S-phase population in D as compared to C is visible in the plots. See table 1 for details.

We have previously established a protocol of siRNA-mediated knockdown of CGGBP1 at both mRNA and protein levels [3,4]. In this study, we used the same protocol of siRNA mediated knockdown of CGGBP1, as described earlier [3,4], to deplete CGGBP1 in the human glioma cell line U-2987 MG. Knockdown by siRNA was efficient as immunofluorescence showed a strong reduction in protein levels 96 h after transfection as compared to control siRNA transfected cells (Figure 1, A and 1B). The cells were transfected at 50% confluence and after 96 h, while control siRNA transfected cultures grew to confluence, CGGBP1 siRNA transfected cultures did not show increase in cell density with no visible difference in the proportion of dead cells, suggesting that the CGGBP1 transfected cells underwent a cell cycle arrest. Expression of the proliferation marker Ki67 was also reduced in the CGGBP1 siRNA transfected cells (Figure 1, A and 1B). Flow cytometric measurement of DNA content per nucleus showed that in the CGGBP1 depleted cultures the G0/G1 population was increased while the population of S phase was strongly decreased (Table 1 and Figure 1, C and 1D). The depletion of CGGBP1 by using the previously described UTR siRNA against CGGBP1 [4] also generated a similar cell cycle arrest in G1/G0 phase with a concomitant decrease in the S phase in U-2987 MG cells stably transfected with an empty expression vector (Additional File 1). However, in U-2987 MG cells stably expressing CGGBP1 from a UTR-devoid transcript, the UTR siRNA failed to produce the G0/G1 arrest (Additional File 1), showing the specificity of siRNA response. These results showed that in the U-2987 MG cells, CGGBP1 is required for normal cell cycle progression and its depletion results in a cell cycle arrest characterized by an accumulation of cells in the G0/G1 phase and a parallel reduction in the S-phase population.

**Table 1:** Effect of CGGBP1 depletion in different cell types on the percentage of cells in different stages of cell cycle as measured by flow cytometric analysis of DNA content per nucleus

Cells	siRNA	G1/G0 (% of total)	S (% of total)	G2/M (% of total)
U-2987 MG	Control	84.58 ± 0.16	9.95 ± 0.18	5.44 ± 0.1
	
	CGGBP1	89.68 ± 0.28	2.85 ± 0.04	7.45 ± 0.29

U-2987 MG-vector	Control	64.59 ± 0.72	26.83 ± 0.85	8.57 ± 0.36
	
	UTR	70.03 ± 0.18	22.34 ± 0.35	7.51 ± 0.12

U-2987 MG-CGGBP1	Control	60.82 ± 2.48	28.56 ± 3.04	10.61 ± 0.71
	
	UTR	60.52 ± 2.14	28.46 ± 3.56	11.01 ± 1.49

HCT116 p53+/+;p21+/+	Control	87.12 ± 0.39	11.39 ± 0.05	1.48 ± 0.38
	
	CGGBP1	91.94 ± 0.64	6.88 ± 1.16	1.17 ± 0.51

HCT116 p53-/-;p21+/+	Control	87.46 ± 0.47	10.15 ± 0.44	2.38 ± 0.02
	
	CGGBP1	91.54 ± 0.05	7.66 ± 0.03	0.8 ± 0.04

HCT116 p53+/+; p21-/-	Control	88.44 ± 0.45	10.50 ± 0.39	1.05 ± 0.06
	
	CGGBP1	92.76 ± 0.22	5.55 ± 0.24	1.67 ± 0.02

U2OS	Control	49.82 ± 0.35	37.09 ± 0.45	13.08 ± 0.55
	
	CGGBP1	68.83 ± 0.58	19.29 ± 0.25	11.86 ± 0.47

SAOS2	Control	66.34 ± 0.73	20.58 ± 0.57	12.65 ± 0.50
	
	CGGBP1	75.65 ± 0.49	11.88 ± 0.25	12.46 ± 0.52

WT (Ink4a+/+;Arf+/+)	Control	66.97 ± 0.82	29.85 ± 0.91	3.14 ± 0.12
	
	CGGBP1	75.42 ± 0.58	20.79 ± 0.76	3.78 ± 0.19

Ink4a-/-	Control	71.52 ± 0.61	27.18 ± 0.16	1.29 ± 0.64
	
	CGGBP1	77.91 ± 0.64	17.82 ± 0.36	4.26 ± 0.32

Arf-/-	Control	56.33 ± 1.07	40.13 ± 1.32	3.53 ± 0.62
	
	CGGBP1	68.99 ± 0.78	28.80 ± 0.74	2.19 ± 0.05

U-2987 MG	Control	76.04 ± 0.49	18.75 ± 5.21	5.21 ± 0.32
	
	CGGBP1	84.88 ± 0.60	8.04 ± 7.07	7.07 ± 0.42
	
	GAS1	72.33 ± 0.40	20.22 ± 0.20	7.44 ± 0.60
	
	CGGBP1+ GAS1	92.44 ± 0.32	3.79 ± 0.08	3.76 ± 0.23

N = 3 independent transfections in each case. The differences between the G1/G0 phase and S phase values for control siRNA and CGGBP1 siRNA (or between control siRNA and CGGBP1+GAS1 siRNA combined for the last sample) are significant (Heteroscedastic two tailed T test, p < 0.05). Difference between control siRNA and UTR siRNA is significant for U-2987 MG-vector But insignificant for U-2987 MG-CGGBP1 (Heteroscedastic two tailed T test, p < 0.05). Please note that close to 50% reductions in the S phase populations of most samples due to CGGBP1 knockdown is reflected as a rather minor yet significant increase in the G0/G1 phase populations.

### CGGBP1 deficiency increases the expression of some key cell cycle regulatory genes

**Figure 2:**
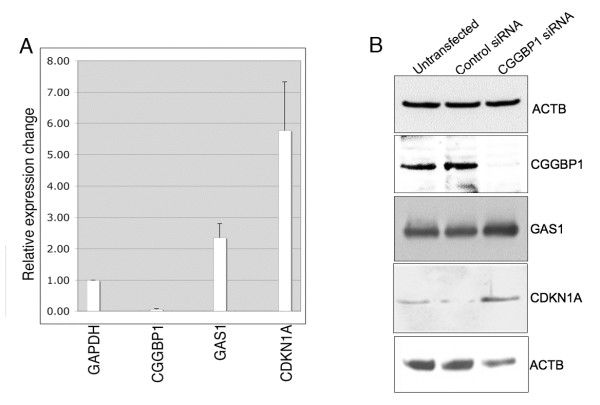
**CGGBP1 depletion in U-2987 MG cells is associated with increased expression of CDKN1A and GAS1**. A: A strong decrease in CGGBP1 mRNA expression shows the efficiency of siRNA transfections. Using GAPDH as control, increase in the mRNA levels of CDKN1A and GAS1 was seen. RNA samples from duplicate transfections were pooled and analyzed in triplicates and relative change in expression calculated by delta delta Ct method. All changes in expression are significant between control siRNA and CGGBP1 siRNA treatments (p < 0.05). Values for control siRNA in each case is normalized to 1 and not depicted. Plotted values are average ± S.D. and significance was calculated using heteroscedastic two-tailed T-test. B: Western blot analyses showed that CDKN1A and GAS1 expression were increased at protein level also. The mRNA levels of TP53 and ARF were also increased, but their protein levels were not increased as detected by western blots (data not shown). ACTB (top panel), CGGBP1 and GAS1 were run on the same blot. ACTB (lowest panel) and CDKN1A were run from same samples on a different blot.

To identify the mechanisms through which CGGBP1 depletion might cause the cell cycle arrest at G0/G1 phase, we focused on the function of CGGBP1 as a transcription-regulatory protein. We tested the possibility if CGGBP1 could regulate expression of key genes coding for proteins involved in cell cycle control at the G0/G1 phase. U-2987 MG cells were transfected with control or CGGBP1 siRNA and relative changes in mRNA levels of a panel of candidate genes including CDKN1A and GAS1 were assayed by quantitative real time RT-PCR (Figure 2A). The mRNA levels of these genes were significantly increased upon CGGBP1 siRNA treatment in assays performed on cDNA samples from three independent transfections. Western blot analysis showed that CDKN1A and GAS1 protein levels were increased upon CGGBP1 depletion (Figure 2B).

CGGBP1 is known to regulate transcription by directly binding to CGG tandem repeats or through its association with other transcription regulatory proteins such as NFIX and HMGN1 [1-3]. Of all the CGGBP1 target genes analyzed, CDKN1A had the strongest increase in expression (Figure 2). Although CDKN1A promoter [23] is CpG rich, it does not contain CGG repeats. However, it contains NFI-binding site and is regulated by NFI proteins including NFIX [23]. However, an *in silico *analysis of promoter sequences (3 Kb upstream and downstream regions from the transcription start site) showed the genomic region harboring the GAS1 gene contains many small interrupted CGG repeats along the entire length of the gene, which contains only one exon and no introns.

Since the increased expression of CDKN1A and GAS1 could be associated with cell cycle arrest in G0/G1, we wanted to understand the mechanisms through which CGGBP1 might regulate expression of CDKN1A and GAS1. To this end, we assayed how CGGBP1 depletion affects the transcription regulatory chromatin profiles at GAS1 and CDKN1A promoters.

### CGGBP1 binds to and affects chromatin profiles at the CDKN1A and GAS1 promoters

**Figure 3:**
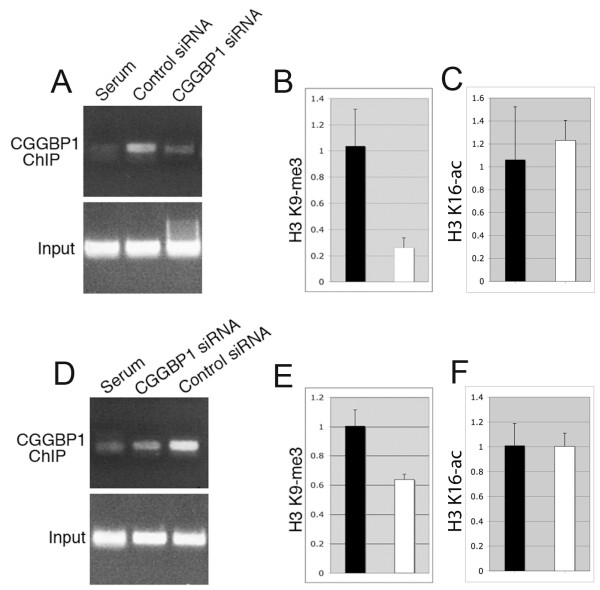
**CGGBP1 regulates H3K9 trimethylation levels at CDKN1A and GAS1 promoters**. A: CGGBP1 binds to CDKN1A promoter and this is sensitive to CGGBP1 siRNA, thus showing the specificity of the assay. B: H3K9 trimethylation is reduced at the CDKN1A promoter after CGGBP1 depletion whereas as shown in C, H3K16 acetylation was not significantly changed. D: CGGBP1 binds to GAS1 promoter in a CGGBP1 siRNA-sensitive manner. E: H3K9 trimethylation in GAS1 promoter region is reduced by CGGBP1 depletion whereas the H3K16 acetylation in this region was unaffected by CGGBP1 depletion (F). For exact region details of the assayed region of CDKN1A and GAS1 promoters, see primers mentioned in materials and methods. ChIP DNA samples from 3 independent assays were pooled and analyzed in triplicates. Input DNA was used to normalize the amount of DNA and used as baseline in double delta Ct calculations to find out relative changes in DNA-protein interactions. Changes in real-time PCR are significant (p < 0.05, heteroscedastic two-tailed T-test) in B and E only. For B, C, E and F, the black bars represent the control siRNA treated samples and the white bars represent CGGBP1 siRNA treated samples.

Chromatin immunoprecipitation (ChIP) assays showed that CGGBP1 was associated with the CDKN1A promoter (Figure 3A). Histone H3K9 trimethylation, a transcription repressive histone modification, was reduced at the CDKN1A promoter in CGGBP1 depleted samples compared to the control siRNA (Figure 3B). ChIP-quantitative PCRs (qPCRs) showed that CGGBP1 depletion did not affect the level of histone H3K16 acetylation at the CDKN1A promoter (Figure 3C).

ChIP assays for CGGBP1 binding to the flanking regions of the GC-rich GAS1 gene showed that CGGBP1 was recruited to this locus (Figure 3D). H3K9 trimethylation was significantly reduced by CGGBP1 depletion (Figure 3E). However CGGBP1 depletion did not affect H3K16 acetylation in this region (Figure 3F).

These results showed that CGGBP1 maintains a specific H3K9 trimethylation code at the CDKN1A and GAS1 promoters, which is disturbed after its depletion and is associated with their increased expression.

### The G0/G1 arrest caused by CGGBP1 siRNA does not depend singly on TP53, CDKN1A, INK4A, ARF or GAS1 expression

We then tested if the expression of the above mentioned cell cycle regulatory genes, is required for CGGBP1 siRNA-induced G0/G1 arrest. We performed cell cycle analyses in cells of different genetic backgrounds transfected with control or CGGBP1 siRNA. For all the batches of siRNA transfections, CGGBP1 knockdown was confirmed by real time qRT-PCR (not shown).

**Figure 4:**
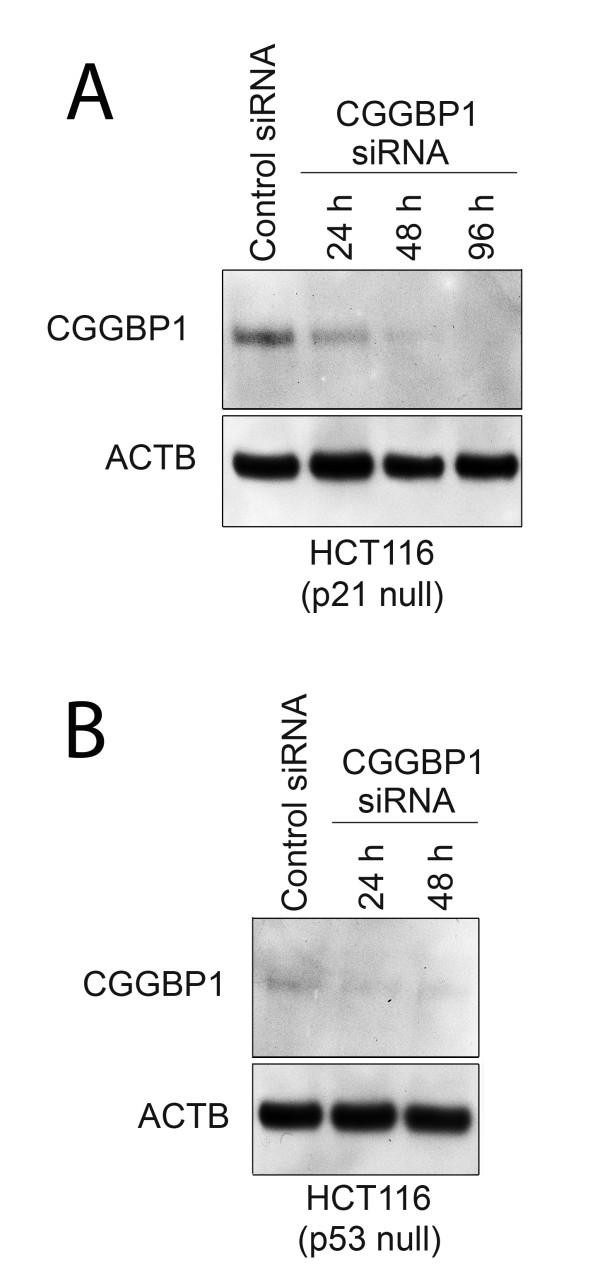
**CGGBP1 siRNA causes CGGBP1 depletion in HCT116 cells**. CGGBP1 siRNA causes CGGBP1 depletion in HCT116 cells deficient for CDKN1A (p21) (A) or TP53 (p53) (B). Different time points after siRNA transfections are indicated on the top of each lane. ACTB expression was used as loading control.

First, we asked if the increased level of CDKN1A is the key event underlying the CGGBP1 siRNA-induced G0/G1 arrest. We compared the effects of CGGBP1 siRNA in HCT116 human colon cancer cell lines either WT or deficient for CDKN1A. CGGBP1 depletion in HCT116 cells null for CDKN1A was additionally confirmed by western blotting at different time points (Figure 4A). CGGBP1 siRNA caused G0/G1 arrest in the cells irrespective of the CDKN1A genotype showing that CDKN1A is not required for CGGBP1 depletion-induced cell cycle arrest (Table 1 and Additional Files 2 and 3). Similar assay comparing WT or TP53 null HCT116 cells (Figure 4B) showed that even TP53 deficiency alone could not rescue CGGBP1 siRNA induced cell cycle arrest as both WT and TP53 null cells showed a similar G0/G1 arrest (Table 1 and Additional Files 2 and 4). Transfection of control or CGGBP1 siRNA into U2OS (TP53 WT) and SAOS2 (TP53 deficient) cells showed that although the G0/G1 arrests were stronger in U2OS than in SAOS2, it did occur in both the cell lines (Table 1 and Additional Files 5 and 6). These results suggested that TP53 and CDKN1A are not single determinants of cell cycle regulation by CGGBP1.

We then investigated if the cell cycle inhibitors INK4A and ARF are required for CGGBP1 depletion induced G0/G1 arrest. Using the RCAS/TV-A system [24] we established mouse glioma cell lines, derived from PDGFB induced tumors, in Ntv-a (the avian TVA receptor for RCAS virus driven by Nestin promoter) transgenic mice with wild type, Ink4A-/- or Arf-/- genetic backgrounds. One cell line from each genotype was tested for the effects of control or CGGBP1 siRNA on their cell cycle patterns. A G0/G1 arrest with reductions in S phase population was seen in all the three cell lines (Table 1 and Additional Files 7, 8 and 9). These results established that INK4A and ARF are not the master regulators of CGGBP1 depletion induced G0/G1 arrest.

Finally we tested if knocking down the GAS1 levels in CGGBP1 depleted cells would rescue the CGGBP1 siRNA-induced cell cycle arrest. Equimolar amounts of control siRNA, CGGBP1 siRNA, GAS1 siRNA or a 1:1 molar cocktail of CGGBP1 and GAS1 siRNA were transfected into U-2987 MG cells and later analyzed by flow cytometry. Like CGGBP1, GAS1 knockdown was confirmed by real time qRT-PCR (not shown). As compared to the control siRNA, CGGBP1 siRNA produced a G0/G1 arrest as before (Table 1 and Additional File 10), whereas the GAS1 siRNA alone produced a mild but insignificantly increased flux through S phase and a decrease in the G0/G1 population was seen (Table 1 and Additional File 10). However, a combination of GAS1 and CGGBP1 siRNA failed to rescue the G0/G1 arrest. Surprisingly, we saw a further increase in the G0/G1 population and stronger reductions in the S- and G2/M phases (Table 1 and Additional File 10). These results suggested that GAS1 is not the key regulator of CGGBP1 siRNA-induced cell cycle arrest and that the increased efflux of cells from G0/G1 phase into the S-phase caused by GAS1 knockdown is unsustainable in the absence of CGGBP1.

## Discussion

Regulation of cell cycle through the G1 phase transition has been a hitherto unknown function of CGGBP1. Our previous work has shown that CGGBP1, a transcription regulatory protein, participates in heat shock and related stress responses [3], is a midbody protein with AURKB-like expression and is involved in abscission [4].

Of all the previously known functions of CGGBP1, its role in transcription [2] has been evaluated here for the G0/G1 arrest phenotype observed in cancer cells in this study. Our results show that transcription regulation of cell cycle regulatory genes is a mechanism through which CGGBP1 might affect the cell cycle progression. While we found increases in the transcript levels of TP53 (not shown), CDKN1A, GAS1 and ARF (not shown) genes after CGGBP1 depletion, we addressed the mechanism of expression regulation only for CDKN1A and GAS1 genes as only for these genes the changes in transcript levels were correlated with a change in protein levels. We tested the histone acetylation and methylation profile of the CDKN1A and GAS1 promoters and found that H3K9 trimethylation, a well-established transcription repressive histone modification [25,26] was reduced and this reduction could well explain the increase in CDKN1A transcript levels. CGGBP1 is known to require CGG triplet repeats to bind to the DNA and execute its transcriptional regulatory functions. However, CDKN1A promoter in the present study has turned out to be a CGG-repeat-free region, which exhibits binding to CGGBP1 and undergoes changes in H3K9 trimethylation upon CGGBP1 depletion. This shows that CGG repeats are not absolutely required for CGGBP1 binding and activity. H3K9 trimethylation could induce compaction of the chromatin and recruit chromobox domain containing proteins, which can in turn recruit transcription repressors to the locus [25,26]. H3K9 trimethylation can also result from and lead to CpG methylation of the DNA interestingly, the region around the CDKN1A transcription site is GC-rich and could be subjected to changes in CpG methylation. Similar observations about the changes in the histone modifications were also made at the GAS1 locus, which is rich in small interrupted CGG repeats. While the levels of H3K16 acetylation were unaffected, H3K9 trimethylation were decreased. These results suggest that the control of H3K9 trimethylation is a mechanism by which CGGBP1 manifests its effects on transcriptional regulation of different genes, including CDKN1A and GAS1. Interestingly, CGGBP1 has been reported to be a binding partner of SUV39H2 [27]. SUV39H2 has been shown to a member of the family of histone methyltransferases and can control histone H3K9 trimethylation levels [28]. How CGGBP1 affects SUV39H2 binding to the DNA and affects its H3K9 tri-methylation activity will be an interesting topic for future work.

We could address the effects of the functional deficiencies of TP53, CDKN1A, GAS1, INK4A and ARF genes on CGGBP1 siRNA-induced G0/G1 arrest by testing the deficiencies of only one gene at a time, while the increased expression of these genes occur simultaneously upon CGGBP1 depletion. Our results show that none of these genes are single master regulators of the cell cycle regulatory events downstream of CGGBP1. Due to the complexity of the signal transduction pathways, it is difficult to predict if a combined functional deficiency of these genes would rescue the effects of CGGBP1 siRNA on cell cycle progression or not. Our conclusions are also confounded by the fact that the genotypes of the different cell types used in this study, at all major cell cycle regulatory genes, is not known and to establish them is beyond the scope of this study. Nevertheless, using the matched background genotypes of HCT116 cells, widely used P53 null and WT pairs of SAOS2 and U2OS and combinations of siRNAs against CGGBP1 and GAS1, we can safely conclude that of TP53, CDKN1A, GAS1, INK4A and ARF, none alone is a critical master regulator of the G0/G1 arrest induced by CGGBP1 depletion. These results underscore the fact that although multiple checkpoint escape mechanisms exist in different cancer cells, ablation of CGGBP1 function compromises their ability to escape from the mechanisms blocking cell cycle in G0/G1 phase. CGGBP1 thus regulates cell cycle through multiple parallel mechanisms and further studies on its mechanisms of action will be important in understanding how cancer cell proliferation could be controlled.

## Conclusions

CGGBP1 is required for the ability of cancer cells to progress cell cycle beyond G0/G1 phase even if they have single deficiencies of functional TP53, CDKN1A, INK4A, ARF and GAS1 genes. This shows that the absence of CGGBP1 overrides the ability of cancer cells to escape the cell cycle block at G0/G1 phase, conferred by lack of the above mentioned cell cycle regulatory genes. CGGBP1 seems to regulate the passage through G0/G1 phase through multiple parallel mechanisms. Our results necessitate investigations into expression and function of CGGBP1 in cancers.

## Methods

### Cell culture and siRNA transfections

U-2987 MG cells were previously described as the cell line number 18 by Hagerstrand and co-workers [29]. U-2987 MG cells were cultured in Eagle's minimum essential medium (SIGMA). Human CGGBP1 siRNA, UTR siRNA and control siRNA (Dharmacon) have been described before [3,4]. All siRNA transfections were done using Dharmafect 2 transfection reagent. The molarity of siRNA was equal between different samples compared against each other for all the assays (200 nM for the samples included in the assays involving GAS1 knockdown and 100 nM for all other assays). Mouse CGGBP1 siRNA (catalogue number L-057812-01), human GAS1 siRNA (catalogue number L-011665-00) and control siRNA (catalogue number D-001810-10) were from Dharmacon, ThermoScientific.

### Immunofluorescence

Ki67 and CGGBP1 were detected by immunofluorescence using a mouse monoclonal anti-human Ki67 (DAKO) and a rabbit polyclonal anti-human CGGBP1 (GenTex). The cells were transfected with siRNA, 96 h later fixed using 4% formaldehyde in 1 × PBS for 10 minutes, permeabilized using 2% Triton X-100 for 10 minutes and blocked using 10% FCS and 3% BSA in 1 × PBS for 1 h. Primary antibodies were mixed and diluted in 1 × PBS with 0.2% Triton X-100 (Ki67 1:250 and CGGBP1 1:500) and flooded on cells for 1 h at room temperature. Cells were washed with 1 × PBS with 0.2% Triton X-100 for 10 minutes 3 times and incubated with a mixture of secondary antibodies (FITC-donkey anti-rabbit from Invitrogen, 1:500 and RRX-donkey anti-mouse from Jackson Labs, 1:400) for 1 h. Washings were performed with 1 × PBS with 0.2% Triton X-100 for 10 minutes, 3 times and cells were mounted with a DAPI containing aqueous mounting medium. Cells were analyzed with a Zeiss Meta confocal microscope.

### Western blot analysis

Western blot analyses were performed using 4-12% Bis-Tris gels of NuPAGE electrophoresis system from Invitrogen. The gels were run using MES or MOPS buffers (Invitrogen). Primary antibodies were as follows: ACTB (SIGMA), CGGBP1 (ABCAM), GAS1 (GenTex), CDKN1A (Roche). Semi-dry transfers were performed on nitrocellulose membranes (GE Life Sciences) using NuPAGE transfer buffer (Invitrogen). All HRP-conjugated secondary antibodies were from GE Life Sciences and chemiluminescent substrate for HRP was from Pierce.

### Real time quantitative RT-PCRs (q-RTPCR)

Real time q-RTPCRs were performed using the SYBR Green master mix from Applied Biosystems. RNA from pooled cells from double assays was extracted from siRNA treated cells and cDNA was made from 2 μg of total RNA using reverse transcription reagents from New England Biolabs in a 100 μl reaction mix. The cDNA was diluted to 1 ml and 10 μl was used as a template for each assay. Real time assays were run on a Stratagene PCR machine and melting curve was used to assure specific amplification of PCR products. Results were analyzed using double delta Ct method. The Ct values for GAPDH as a control for the amount of RNA were used for first delta Ct deduction and the delta-Ct values from control siRNA were used for the second delta delta Ct deductions. All assays were performed in triplicates and results plotted as average ± S.D. Significance was calculated by performing heteroscedastic two-tailed T-test in Excel. The primers were: CDKN1A (ATGAAATTCACCCCCTTTCC and CCCTAGGCTGTGCTCACTTC), TP53 (GGCCCACTTCACCGTACTAA and GTGGTTTCAAGGCCAGATGT), INK4A (ACCGGAGGAAGAAAGAGGAG and CGTAACTATTCGGTGCGTTG), ARF (AGTTAAGGGGGCAGGAGTG and GGCTCCTCAGTAGCATCAGC) and GAS1 (CGGAGCTTGACTTCTTGGAC and CCCAACCCTTCAAATTGCTA). Primers for CGGBP1 and GAPDH RTPCRs have been described elsewhere [3].

### Cell cycle analysis by flow cytometry

For cell cycle analysis by flow cytometry, propidium iodide staining of nuclei was performed as described earlier [30,31]. Stained nuclei were analyzed on a BD Biosciences flow cytometer and result files analyzed using automatic settings in the ModFit program. Assays were performed on three independent and parallel transfections and the values of 2N (G0/G1 phase), 4N (G2/M phase) and intermediate (S phase) populations was averaged and significance calculated using heteroscedastic two-tailed T-test in Excel.

### Chromatin immunoprecipitation assays and quantitative PCRs

Chromatin immunoprecipitation (ChIP) assays and quantitative PCRs on ChIP DNA were performed as described earlier [3]. For CDKN1A ChIP assays the chromatin was sheared to a size range of < 500 base pairs. For GAS1 ChIP assays the chromatin was sheared to a size range of between 500 base pairs and 1 Kb and the PCR mixes were supplemented with DMSO to a final concentration of 10% v/v. The changes shown in Figure 3A, B and 3C are using the primers ACTGGGGGAGGAGGGAAG and GCGGCCCTGATATACAACC in CDKN1A promoter. Using further upstream primers (CTCTCCAATTCCCTCCTTCC and AGAAGCACCTGGAGCACCTA), or further downstream primers (AGCGGAGTGGAGTAAGTTCG and TCACCTCCTCGCTAGTCCTT) the enrichment using CGGBP1 antibody versus serum control was very low as compared to the results shown in A (data not shown). The results shown in C, D and F are using the primer pair GCGGGTTGTAAGCATCTCAT and CATCTGTGCTTTCGACTGGA. Primers closer to the GAS1 transcription start site did not work likely due to the high CGG richness of the DNA in that region. All primer sequences are from 5'end to the 3'end of the synthesized oligonucleotides.

### Generation of mouse glioma cell cultures

Mouse glioma cell cultures were established from PDGF-B induced gliomas in Ntv-a wildtype, Ink4a-/- or Arf-/- mice [32,33]. Mouse brain tumors were generated by intracerebral injection of 5 μl of RCAS-PDGFB-IRES-eGFP producing DF-1 chicken fibroblasts into newborn mice. The injected mice were sacrificed when showing any sign of sickness, but at the latest at 12 weeks of age. The brains were collected under aseptic conditions and a coronal section was made at the injection site and one part was collected and embedded in paraffin post formalin fixation or snap-frozen and embedded in OCT, whereas the other part was minced and dissociated for culturing. The mouse cell lines were cultured in Dulbecco's Modified Eagle's Medium (DMEM, Sigma Aldrich) supplemented with 10% fetal bovine serum (FBS, Gibco), 4 mM L-glutamine and 100 units/ml penicillin and 0.1 mg/ml streptomycin (Sigma Aldrich). All cells were grown at 37°C with 5% CO2. All animal experiments were approved by the "Ethical Committee for Animal Experiments in Uppsala (Sweden)". The ethical approval number for these animal experimentations from the Swedish Agriculture Board was C18/6.

## Authors' contributions

US performed the experiments analyzed the results and drafted the manuscript, PR generated the mouse glioma cell lines, LU supervised the generation of mouse cell lines by PR, BW supervised the overall project, analyzed the results and helped in drafting the manuscript. All authors read and approved the final manuscript.

## Supplementary Material

Additional file 1 **Flow cytometric pattern of U-2987 MG cells stably selected for containing an empty vector (PCDNA3.1+) or CGGBP1-expressing vector, transfected with control or UTR siRNA**. The CGGBP1 UTR siRNA-induced decrease in S phase and an increase in the G1/G0 phase cell population in U-2987 MG cells is rescued by the expression of a UTR-free CGGBP1 cDNA and not by the empty vector alone. This proves the specificity of the effect of the siRNA. Click here for file

Additional file 2 **Flow cytometric pattern of HCT116 (p21 wt and p53 wt) cells treated with control or CGGBP1 siRNA**. CGGBP1 siRNA produces a decrease in S phase and an increase in the G1/G0 phase cell population in HCT116 cells with wild-type p21 and p53 genes. Click here for file

Additional file 3 **Flow cytometric pattern of HCT116 (p21 null) cells treated with control or CGGBP1 siRNA**. CGGBP1 siRNA produces a decrease in S phase and an increase in the G1/G0 phase cell population in HCT116 cells with mutant p21 and wild-type p53 genes. Click here for file

Additional file 4 **Flow cytometric pattern of HCT116 (p53 null) cells treated with control or CGGBP1 siRNA**. CGGBP1 siRNA produces a decrease in S phase and an increase in the G1/G0 phase cell population in HCT116 cells with wild-type p21 and mutant p53 genes. Click here for file

Additional file 5 **Flow cytometric pattern of SAOS2 cells treated with control or CGGBP1 siRNA**. CGGBP1 siRNA produces a decrease in S phase and an increase in the G1/G0 phase cell population in SAOS2 cells. These cells are known to be p53 deficient. Click here for file

Additional file 6 **Flow cytometric pattern of U2OS cells treated with control or CGGBP1 siRNA**. CGGBP1 siRNA produces a decrease in S phase and an increase in the G1/G0 phase cell population in U2OS cells. These cells are known to have wild-type p53. Click here for file

Additional file 7 **Flow cytometric pattern of mouse glioblastoma cells (INK4A WT and ARF WT) treated with control or CGGBP1 siRNA**. Mouse CGGBP1 siRNA produces a decrease in S phase and an increase in the G1/G0 phase cell population in PDGFB-overexpressing mouse glioma cell lines which are wild-type for INK4A and ARF. Click here for file

Additional file 8 **Flow cytometric pattern of mouse glioblastoma cells (INK4A-/-) treated with control or CGGBP1 siRNA**. Mouse CGGBP1 siRNA produces a decrease in S phase and an increase in the G1/G0 phase cell population in PDGFB-overexpressing mouse glioma cell lines which are mutant for INK4A and wild-type for ARF. Click here for file

Additional file 9 **Flow cytometric pattern of mouse glioblastoma cells (ARF-/-) treated with control or CGGBP1 siRNA**. Mouse CGGBP1 siRNA produces a decrease in S phase and an increase in the G1/G0 phase cell population in PDGFB-overexpressing mouse glioma cell lines which are mutant for INK4A and wild-type for ARF. Click here for file

Additional file 10 **Flow cytometric pattern of U-2987 MG cells treated with control, CGGBP1, GAS1 or CGGBP1+GAS1 siRNA**. While the CGGBP1 siRNA produces a decrease in S phase and an increase in the G1/G0 phase cell population in U-2987 MG cells, GAS1 siRNA caused increased proliferation, represented by an increased flux of cells into the S phase. Combination of CGGBP1 and GAS1 siRNA showed that in the absence of CGGBP1, the increased S-phase population of cells caused by GAS1 depletion is unsustainable. The combination of the two siRNA thus caused a synergistic and stronger G1/G0 arrest and decrease in the S phase population. Click here for file
