# Supplementary figures and images for: CGGBP1 regulates cell cycle in cancer cells
Source: BMC Mol Biol. 2011 Jul 7;12:28. doi: 10.1186/1471-2199-12-28 (PMC3142506; doi:10.1186/1471-2199-12-28)

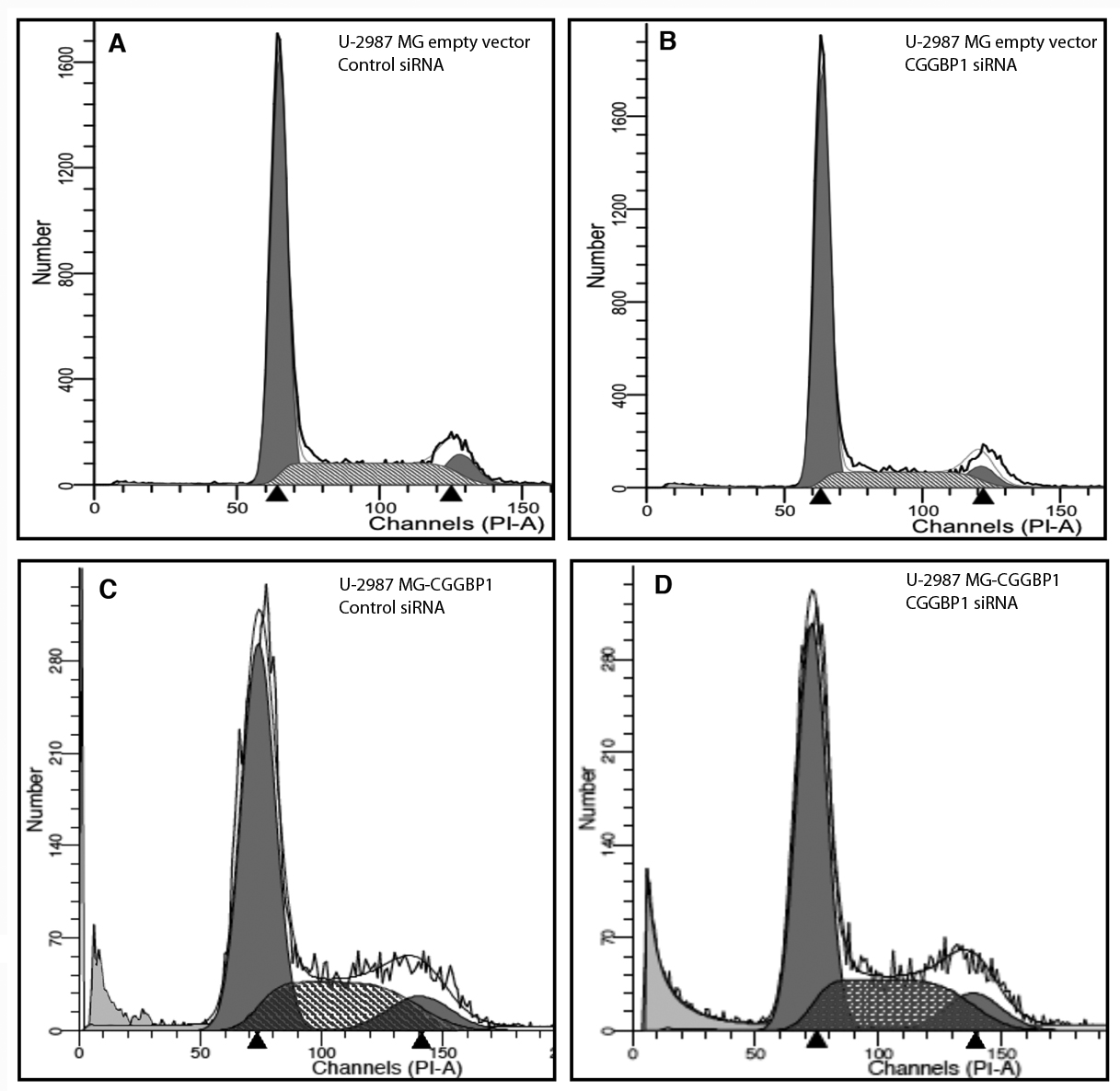

Supplement: Additional file 1 — Flow cytometric pattern of U-2987 MG cells stably selected for containing an empty vector (PCDNA3.1+) or CGGBP1-expressing vector, transfected with control or UTR siRNA. The CGGBP1 UTR siRNA-induced decrease in S phase and an increase in the G1/G0 phase cell population in U-2987 MG cells is rescued by the expression of a UTR-free CGGBP1 cDNA and not by the empty vector alone. This proves the specificity of the effect of the siRNA. [file 1471-2199-12-28-S1.JPEG]

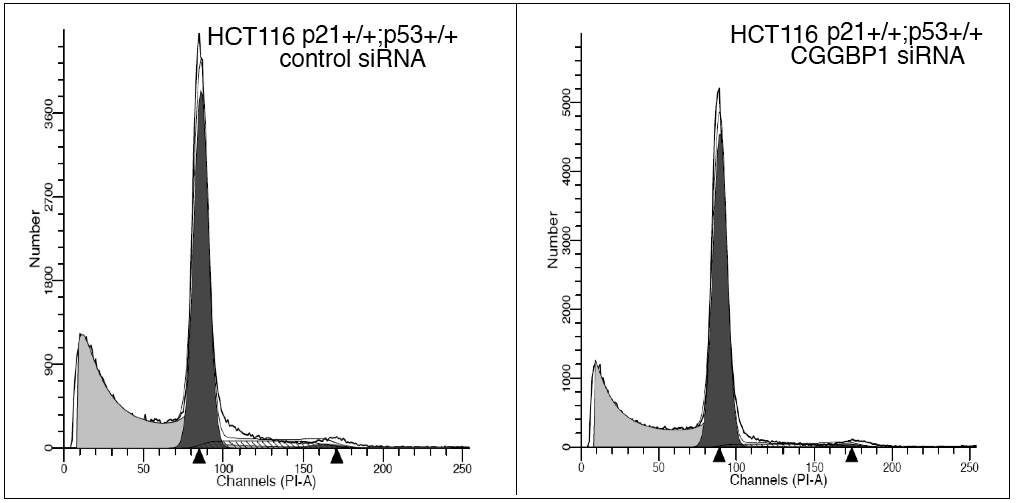

Supplement: Additional file 2 — Flow cytometric pattern of HCT116 (p21 wt and p53 wt) cells treated with control or CGGBP1 siRNA. CGGBP1 siRNA produces a decrease in S phase and an increase in the G1/G0 phase cell population in HCT116 cells with wild-type p21 and p53 genes. [file 1471-2199-12-28-S2.JPEG]

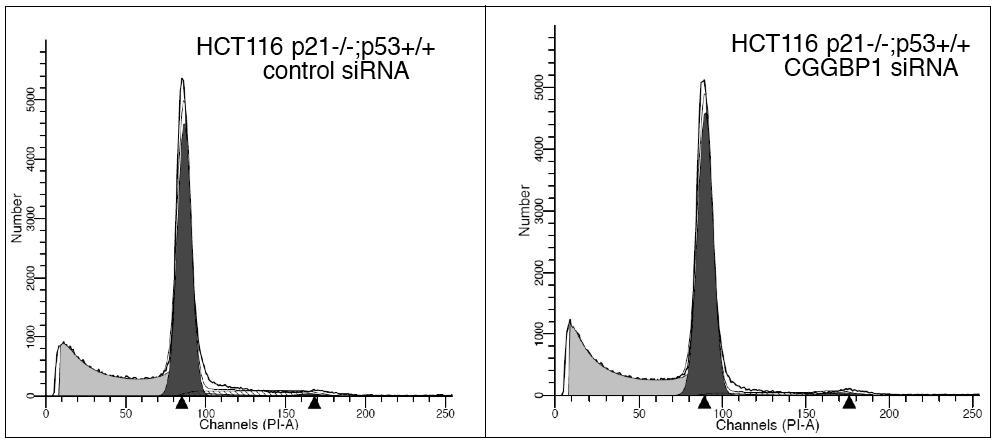

Supplement: Additional file 3 — Flow cytometric pattern of HCT116 (p21 null) cells treated with control or CGGBP1 siRNA. CGGBP1 siRNA produces a decrease in S phase and an increase in the G1/G0 phase cell population in HCT116 cells with mutant p21 and wild-type p53 genes. [file 1471-2199-12-28-S3.JPEG]

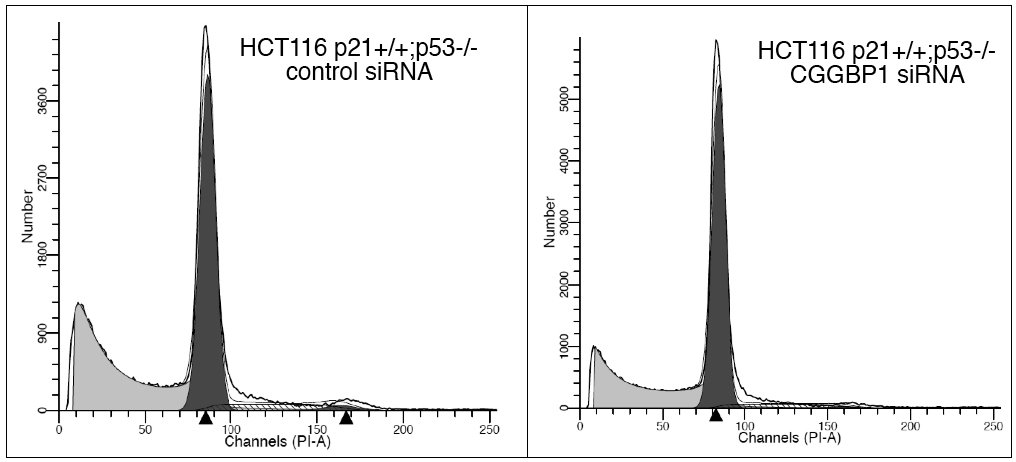

Supplement: Additional file 4 — Flow cytometric pattern of HCT116 (p53 null) cells treated with control or CGGBP1 siRNA. CGGBP1 siRNA produces a decrease in S phase and an increase in the G1/G0 phase cell population in HCT116 cells with wild-type p21 and mutant p53 genes. [file 1471-2199-12-28-S4.JPEG]

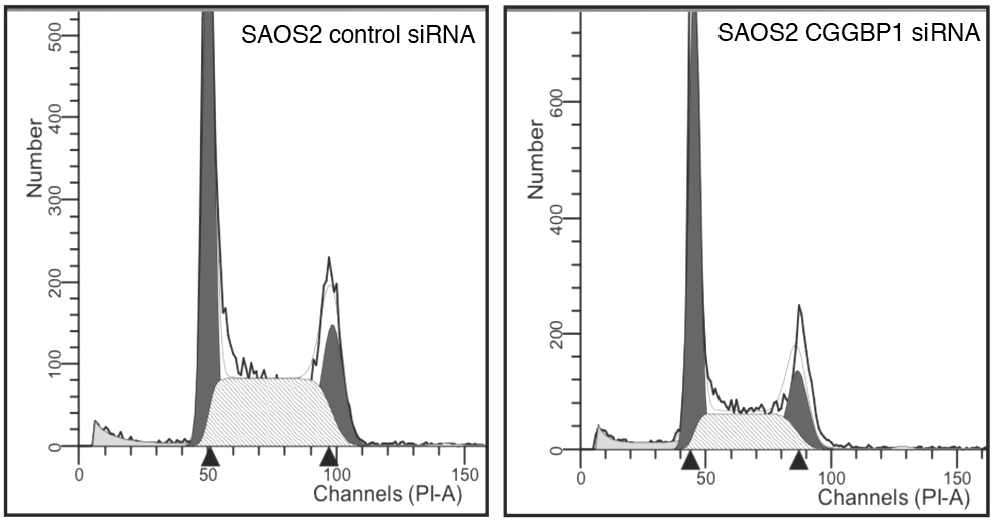

Supplement: Additional file 5 — Flow cytometric pattern of SAOS2 cells treated with control or CGGBP1 siRNA. CGGBP1 siRNA produces a decrease in S phase and an increase in the G1/G0 phase cell population in SAOS2 cells. These cells are known to be p53 deficient. [file 1471-2199-12-28-S5.JPEG]

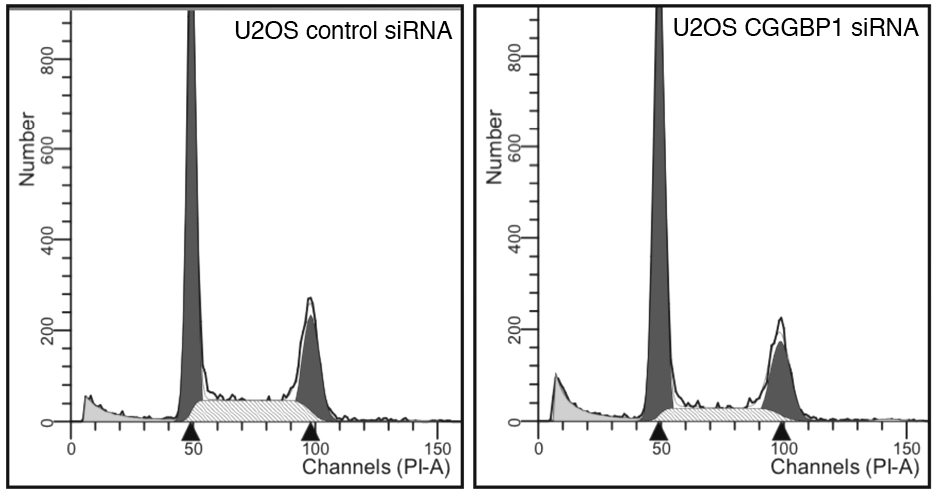

Supplement: Additional file 6 — Flow cytometric pattern of U2OS cells treated with control or CGGBP1 siRNA. CGGBP1 siRNA produces a decrease in S phase and an increase in the G1/G0 phase cell population in U2OS cells. These cells are known to have wild-type p53. [file 1471-2199-12-28-S6.JPEG]

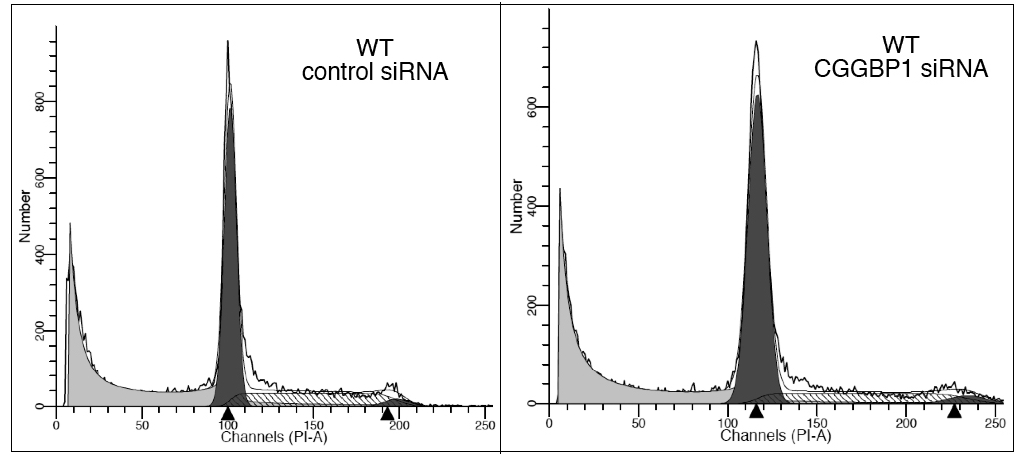

Supplement: Additional file 7 — Flow cytometric pattern of mouse glioblastoma cells (INK4A WT and ARF WT) treated with control or CGGBP1 siRNA. Mouse CGGBP1 siRNA produces a decrease in S phase and an increase in the G1/G0 phase cell population in PDGFB-overexpressing mouse glioma cell lines which are wild-type for INK4A and ARF. [file 1471-2199-12-28-S7.JPEG]

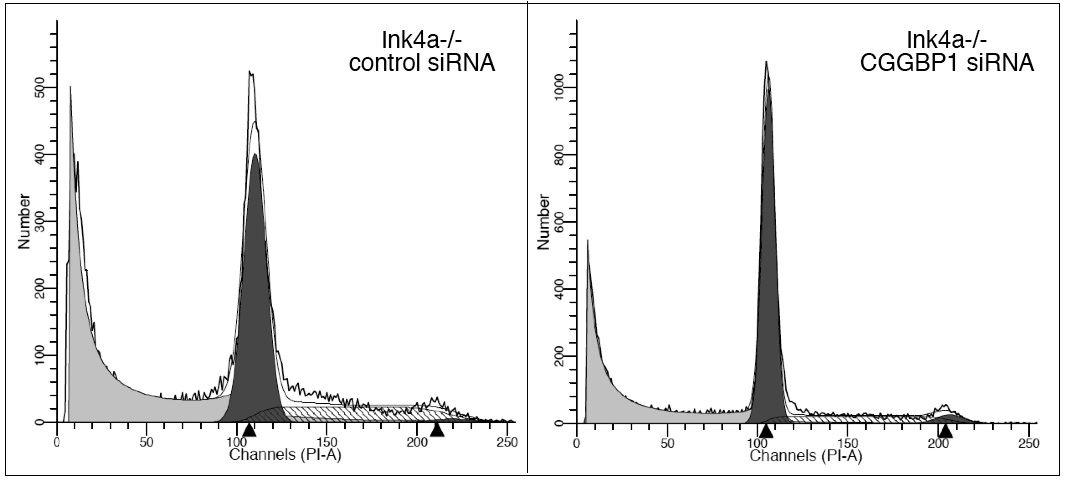

Supplement: Additional file 8 — Flow cytometric pattern of mouse glioblastoma cells (INK4A-/-) treated with control or CGGBP1 siRNA. Mouse CGGBP1 siRNA produces a decrease in S phase and an increase in the G1/G0 phase cell population in PDGFB-overexpressing mouse glioma cell lines which are mutant for INK4A and wild-type for ARF. [file 1471-2199-12-28-S8.JPEG]

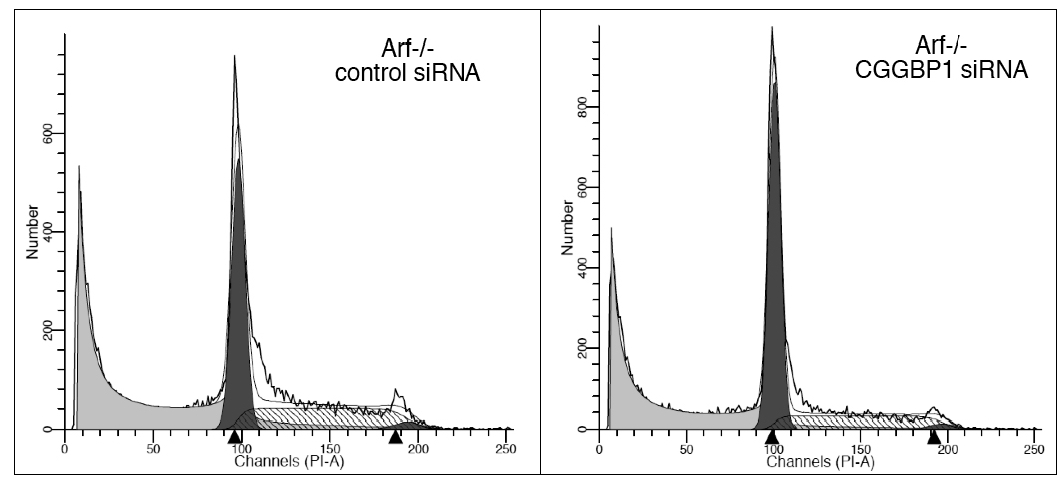

Supplement: Additional file 9 — Flow cytometric pattern of mouse glioblastoma cells (ARF-/-) treated with control or CGGBP1 siRNA. Mouse CGGBP1 siRNA produces a decrease in S phase and an increase in the G1/G0 phase cell population in PDGFB-overexpressing mouse glioma cell lines which are mutant for INK4A and wild-type for ARF. [file 1471-2199-12-28-S9.JPEG]

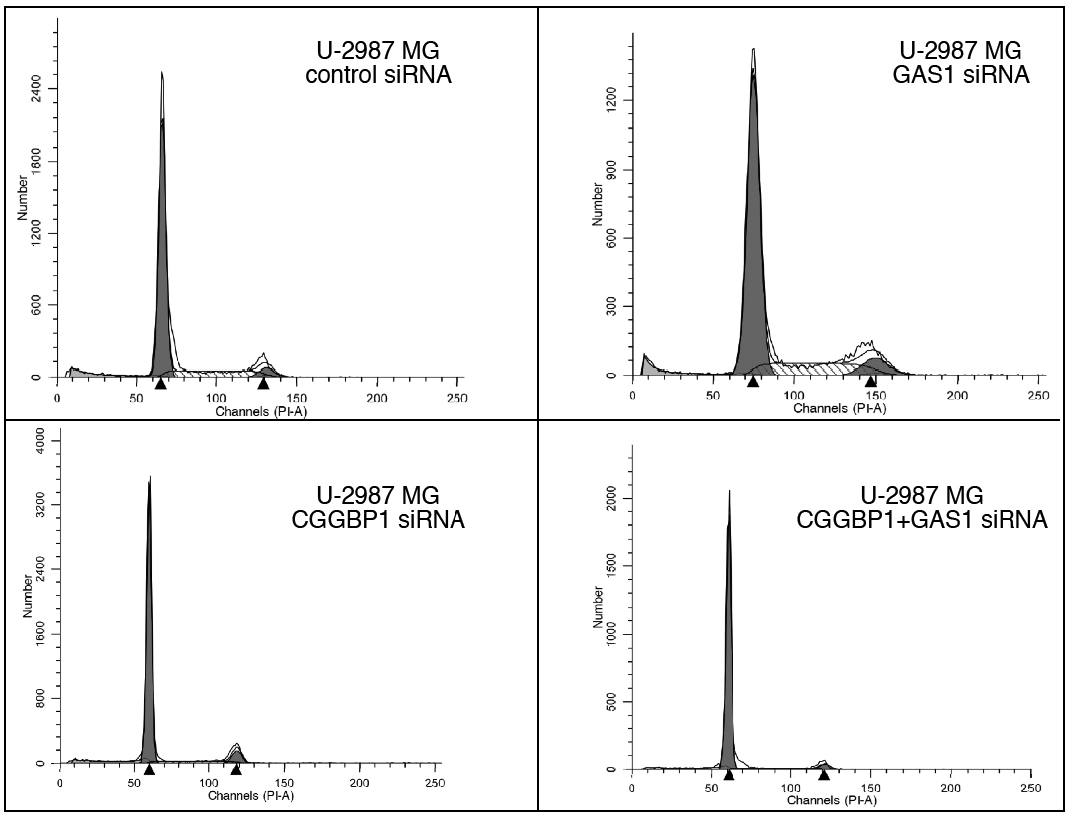

Supplement: Additional file 10 — Flow cytometric pattern of U-2987 MG cells treated with control, CGGBP1, GAS1 or CGGBP1+GAS1 siRNA. While the CGGBP1 siRNA produces a decrease in S phase and an increase in the G1/G0 phase cell population in U-2987 MG cells, GAS1 siRNA caused increased proliferation, represented by an increased flux of cells into the S phase. Combination of CGGBP1 and GAS1 siRNA showed that in the absence of CGGBP1, the increased S-phase population of cells caused by GAS1 depletion is unsustainable. The combination of the two siRNA thus caused a synergistic and stronger G1/G0 arrest and decrease in the S phase population. [file 1471-2199-12-28-S10.JPEG]
